# Multi-Organs-on-Chips: Towards Long-Term Biomedical Investigations

**DOI:** 10.3390/molecules24040675

**Published:** 2019-02-14

**Authors:** Yi Zhao, Ranjith Kumar Kankala, Shi-Bin Wang, Ai-Zheng Chen

**Affiliations:** 1 Institute of Biomaterials and Tissue Engineering, Huaqiao University, Xiamen 361021, China; 1526221050@hqu.edu.cn (Y.Z.); sbwang@hqu.edu.cn (S.-B.W.); 2 Fujian Provincial Key Laboratory of Biochemical Technology (Huaqiao University), Xiamen 361021, China

**Keywords:** long-term testing, multi-organ-on-chip, microfluidic technology, biosensors, multisensor-integrated systems, drug testing, disease modeling

## Abstract

With advantageous features such as minimizing the cost, time, and sample size requirements, organ-on-a-chip (OOC) systems have garnered enormous interest from researchers for their ability for real-time monitoring of physical parameters by mimicking the in vivo microenvironment and the precise responses of xenobiotics, i.e., drug efficacy and toxicity over conventional two-dimensional (2D) and three-dimensional (3D) cell cultures, as well as animal models. Recent advancements of OOC systems have evidenced the fabrication of ‘multi-organ-on-chip’ (MOC) models, which connect separated organ chambers together to resemble an ideal pharmacokinetic and pharmacodynamic (PK-PD) model for monitoring the complex interactions between multiple organs and the resultant dynamic responses of multiple organs to pharmaceutical compounds. Numerous varieties of MOC systems have been proposed, mainly focusing on the construction of these multi-organ models, while there are only few studies on how to realize continual, automated, and stable testing, which still remains a significant challenge in the development process of MOCs. Herein, this review emphasizes the recent advancements in realizing long-term testing of MOCs to promote their capability for real-time monitoring of multi-organ interactions and chronic cellular reactions more accurately and steadily over the available chip models. Efforts in this field are still ongoing for better performance in the assessment of preclinical attributes for a new chemical entity. Further, we give a brief overview on the various biomedical applications of long-term testing in MOCs, including several proposed applications and their potential utilization in the future. Finally, we summarize with perspectives.

## 1. Introduction

Despite the successes and critical advancements in developing various approaches over the past few decades, it is increasingly recognized that the preclinical stages of current drug development pipeline have failed to fulfill the requirements of accurate predictions of drug responses and their extrapolation to humans. Several cell culture systems in vitro are widely used, since they have allowed for more rapid drug discovery studies and disease modeling, and because they provide a controllable environment where cellular growth and activities can be explicitly observed and tested [1,2]. However, conventional 2D culture systems, in which the cells can be cultivated in a monolayer, fail to replicate the biochemical environment in vivo, and other mechanical properties. Moreover, drug diffusion kinetics cannot be demonstrated accurately in 2D cell cultures, where the drug doses are effective in 2D but universally manifest as being ineffective in a real human body, these culture models usually do not maintain their differentiated cell functions [3,4,5,6]. To address the lack of physiological relevance, which is the major drawback of 2D cell cultures, 3D culture models have gained attention with the improved tissue organization and enhanced expression of cell functions [7]. On the other hand, optimal 3D culture models also suffer from a shortcoming of reproducing the characteristics of living organs, which are crucial for their functions, including tissue–tissue interfaces, temporal and spatial gradients of chemicals and oxygen, and the mechanically active microenvironment [3]. To this end, preliminary investigations in vivo using animal models are regarded as the gold standard, and an absolutely necessary step in the drug development process, as they maintain the significant intricacies lying in living systems, evaluate organ–organ crosstalk, and allow for the determination of pharmacological attributes as well as toxicological issues, among others. However, these models also suffer from several limitations, such as the phylogenetic discrepancy between laboratory animals and humans, which makes it difficult to observe and precisely extrapolate from effects and responses on inherently complex interconnected tissues [2,8,9,10]. Therefore, it is increasingly being recognized that preclinical assessments that are based on animal models often end with poor predictions in many cases [11,12]. In addition, several other drawbacks such as the high cost and time, and ethical concerns have all limited the use of animal models as powerful tools for biological and pharmaceutical research [13].

**Figure 1:**
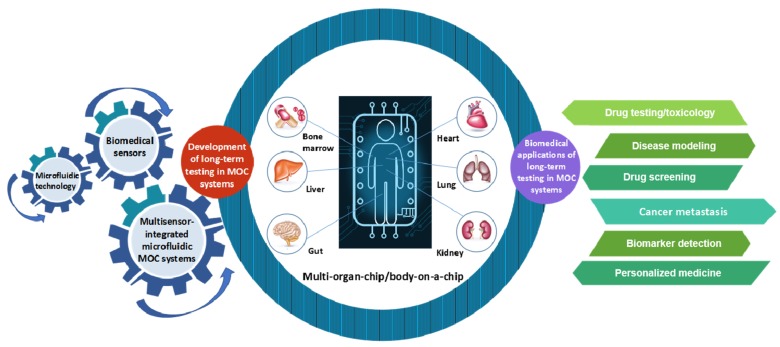
Schematic illustration highlighting the development of long-term testing in MOC systems, and the various proposed and potential biomedical applications of long-term testing in MOC systems.

Recently, organ-on-a-chip (OOC) systems, predominantly based on microfluidic technology, have emerged as alternatives to traditional aforementioned cell culture models, combining cell culture with flow systems that mimic the physiologically relevant conditions and functionalities of organs [14,15,16,17]. Conventionally, numerous OOC models have been fabricated using polydimethylsiloxane (PDMS) elastomer, in which UV lithography has been utilized to create an overall chip architecture, and on the other hand, soft lithography has also been used to generate an imprint of those structures to create microscale fluid channels. In this framework, the PDMS template provides more design flexibility for OOC models, due to its remarkable elasticity. Meanwhile, it can also improve the utilization of normally used optical measuring technologies, and promote their integration with the OOC systems [18,19]. Nevertheless, these models suffer from a few shortcomings, such as the requirements of several labor-intensive steps and specialized equipment, which makes it expensive and hinders rapid iterations of the design, and the difficulty of mimicking the complex structures of the microenvironment in vivo [20]. Recently, 3D bioprinting technology emerged as the most advanced technology for microfluidic device fabrication, and it has been applied to the development of OOC systems due to its processing versatility, rapid generation of microfluidic channels at a high efficiency, user-friendly equipment, and the significant methods that have been developed, using various natural bioinks, bioactive molecules, and cells to construct 3D tissue models in vitro [21,22,23]. 3D bioprinting, usually including stereolithography and extrusion-based printing, creates 3D structures by precisely controlling the spatial distribution, and assembling cells, extracellular matrix (ECM), and other biomaterials layer-by-layer with computer-aided design (CAD) models [20,24,25,26,27]. Based on the characteristics of rapid and continuous model generation, testing, and redesign, 3D bioprinting technology will play a significant role in the fabrication of OOCs with human anatomical as well as physiological features in the future [20,21]. In addition to the benefits of better reflecting the interactions between organs in vivo, OOC approaches generally require much less resources for evaluation, in terms of time and cost [28]. In this framework, various organs that have been significantly replicated and focused on include lungs, liver, blood vessels, intestines, heart, kidneys, and tumor microenvironments [29,30,31,32,33,34,35,36,37,38,39,40,41,42,43]. However, these models are largely based on single-cell types, whose architectures are still far from their respective functional units of organs in the human body. Therefore, over the past years, OOC technology has progressed towards the integration of multiple organ functions on a chip [12,44,45,46,47]. Drugs are generally categorized by the biopharmaceutical classification system (BCS) based on their physical and chemical properties, as well as pharmacokinetic and pharmacodynamic (PK-PD) profiles that result from the complex processes of absorption, distribution, metabolism, and elimination, collectively known as ADME [48,49,50,51,52]. The inaccurate prediction of the PK-PD profile of any drug can increase the failure rate of its development process [53,54]. Thus, it is highly crucial to construct an ideal PK-PD model in order to aid the drug development process [55,56]. Termed as ‘multi-organ-on-chip’ (MOC), sometimes referred as ‘body-on-a-chip (BOC)’, this device combining microscale technology with mathematical PK-PD modeling has separate chambers connected by microfluidic flow channels precisely emulating blood circulation, which provides an approach for monitoring the dynamic responses of multiple organs to pharmaceutical compounds [57]. Despite success in the utilization of MOC systems, there have been several factor in the drug development process that are yet to be addressed, such as the fact that plenty of drugs trigger chronic cellular reactions or induce delayed cell responses. These severe consequences resulted in a number of efforts towards the long-term testing of drugs in MOC systems. Realizing long-term testing using MOC systems can enhance the capability to more accurately and stably detect real chronic cellular reactions in the human body, as well as the interactions of organs in the dynamic ADME process over extended periods of time, which can significantly improve the overall performance of drugs. Despite most of the attention in the field of MOC platforms being on the fabrication of biomimetic multi-organ models, how to realize the long-term investigations using MOCs still remains a significant challenge [3,40,58,59,60,61]. So, in the subsequent sections of this review, we have summarized the recent progress to realize long-term testing using MOCs (Figure 1). First, we describe the basis for utilizing microfluidic technology, highlighting its importance in simulating the circulation system by interconnecting several tissues or organs in the human body. This innovative technology plays an important role in providing a controlled microenvironment for long-term co-culture of multiple tissues in vitro [62]. Second, we emphasize several biomedical sensors as a critical part of achieving long-term and real-time monitoring of multi-organ platforms by measuring microenvironmental parameters (e.g., O_2_, pH) and microelectrode arrays (MEAs) technology, in detecting and recording the electrophysiological responses of organs to xenobiotic compounds. Third, we introduce the utilization of multisensor-integrated microfluidic MOC systems for long-term testing of organoid behaviors. Then, we discuss various biomedical applications of long-term testing in MOCs, including some proposed applications, predominantly focusing on drug testing/toxicology and disease modeling, and the potentials for drug screening, cancer metastasis, biomarker detection, and personalized medicine in the future. Finally, we summarize the different viewpoints and suggest future directions for the MOC field.

## 2. Development of Long-Term Testing in MOC Systems

As mentioned earlier, the long-term testing of MOCs is desired to promote the capability for analyzing multi-organ interactions more accurately and steadily, which bridges the gap between the chronic cellular responses to medications in multi-organ models in vitro, and the real ones in the human body. Herein, we elaborate on the discussion of the efforts to achieve long-term testing in MOC systems and their use with a set of examples.

### 2.1. Advances in Microfluidic Technology for Long-Term Investigations

**Figure 2:**
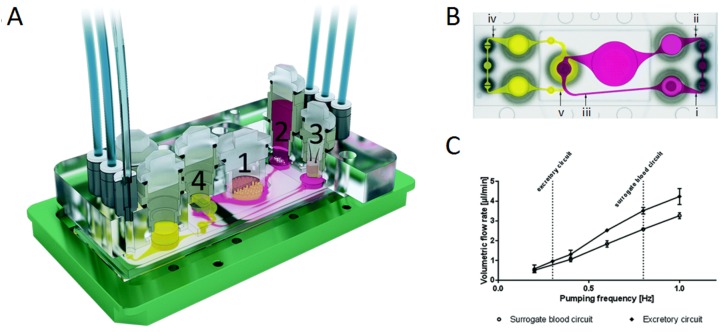
The microfluidic four-organ-chip device at a glance. (**A**) 3D view of the device, comprising two polycarbonate cover-plates, the PDMS-glass chip (footprint: 76 mm × 25 mm; height: 3 mm) accommodating a surrogate blood flow circuit (pink) and an excretory flow circuit (yellow). Numbers represent the four tissue culture compartments for the intestine (1), liver (2), skin (3), and kidneys (4). A central cross-section of each tissue culture compartment aligned along the interconnecting microchannel is depicted. (**B**) Evaluation of fluid dynamics in the 4OC using μPIV (micro-scale particle image velocimetry, an optical method of flow visualization used to obtain instantaneous velocity measurements and related properties in fluids in microscale). Top view of the four-organ-chip layout, illustrating the positions of three measuring spots (i, ii, and iii) in the surrogate blood circuit, and two spots (iv, v) in the excretory circuit. (**C**) Average volumetric flow rate plotted against the pumping frequency of the surrogate blood flow circuit and the excretory circuit. Co-culture experiments were performed at 0.8 Hz and 0.3 Hz, respectively, as indicated by the vertical lines. Error bars are the standard error of the mean. Reproduced from [46], with permission from the Royal Society of Chemistry, 2015.

**Figure 3:**
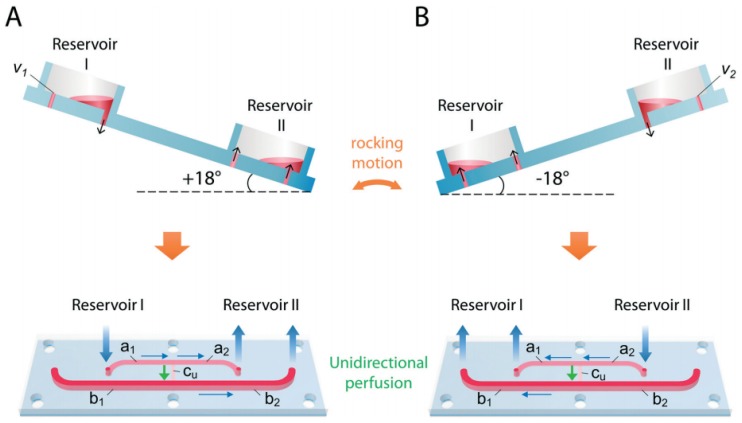
Schematic of UniChip operation. A demonstration UniChip is placed on a rocker platform that flips, tilting between +18° (A) and −18° (**B**) periodically. When tilted at +18° (**A**), flow in b1 is halted by the capillary force at the air–liquid interface in the passive valve v1. Flow is directed from reservoir I through a_1_, a_2_, Cu, and b_2_ into reservoir II. When tilted at −18° (**B**), flow in b_2_ is halted by valve v_2_, and flow is directed from reservoir ii through a_2_, a_1_, Cu and b_1_ into reservoir II. Under either condition, the flow direction in the cell perfusion channel, Cu, is kept the same, as shown by the green arrows. Reproduced from [78], with permission from the Royal Society of Chemistry, 2018.

In traditional 3D cell culture systems, it is extremely difficult to fabricate a testing system with a biomimetic microenvironment to realize functional analyses, the supply of nutrients to cells, trans-cellular transport, removal of cellular by-products, and secretion as well as biochemical analysis of the cultured cells [63,64,65,66]. To fill the gap between in vivo and in vitro conditions, microfluidic approaches have been utilized in OOC technology to simulate organ functions by facilitating the effective transportation [67]. In this framework, the microfluidic flow channels, as well as bioreactors, can provide a steady and sustained flow of culture medium, and connect various organ compartments together for maintaining homeostasis, with which OOC systems have been developed for performing long-term cultivation of cells [68,69,70]. In recent years, several microfluidic perfusion MOCs have been proposed for use in long-term co-cultures of multiple tissues, and continuous observation of the pharmacokinetic ADME process of various drugs. Commonly, traditional perfusion chips use costly and bulky external pumps for the stable flow of the culture medium [71,72]. Micropumps integrated within the chips have now substituted these external pump sources [73,74,75]. In one case, Horland and colleagues constructed an MOC platform equipped with a peristaltic on-chip micropump, interconnecting liver microtissues and skin biopsy culture compartments. This MOC system, providing a controlled medium flow without external media circuits, supported a co-culture of the two tissues for a period of up to 28 days [62]. To further improve upon the fluid dynamics in such multi-organ platforms, Maschmeyer et al. established a four-organ chip for the co-culture of human intestine, liver, skin, and kidney equivalents with two microfluidic circuits. One of the on-chip micropumps assured a near-physiologic fluid flow by interconnecting four tissue culture domains. The second microfluidic flow circuit was used to discharge the liquid secreted by the kidney epithelial cell layer. This microfluidic multi-tissue co-culture device also provided long-term testing of drug candidates over 28 days [46] (Figure 2). However, the utilization of on-chip micropumps is limited, due to the complex manufacturing process, difficulties in integration with the setup, and the requirement of external power supplies for operation, which has resulted in a high demand for simpler alternatives [76]. Accordingly, passively driven perfusion microfluidic MOCs based on gravity-driven perfusion offer the required features, such as a simple design, and have been proposed, as they are inexpensive in manufacturing and operation. In this context, Miller and coworkers designed a pumpless system using a gravity-driven flow system connecting at least 14 chambers for different tissue types. Moreover, they used straight channels across the compartments, and applied the appropriate channel sizes to achieve the optimal flow rates. With the above properties, this approach presented the capability for long-term co-culture of various tissues as well [77]. However, this gravity-induced-perfusion pumpless system circulated fluid bidirectionally, which caused oscillating shear stress, possibly affecting shear stress-sensitive tissues (e.g., vasculature, kidneys, and lungs) [77,78]. Recently, in an attempt to better accommodate shear stress-sensitive tissues, Wang and colleagues utilized the ‘UniChip’ design, which combined specially fabricated supporting channels and passive valves with gravity-driven flow on a BOC platform to achieve recirculating unidirectional perfusion [78] (Figure 3). The results of this study demonstrated that the UniChip design allowed for long-term culture of shear stress-sensitive tissues, and provided a backflow-proof mechanism for the stable chronic operation of BOC systems.

### 2.2. Biomedical Sensors for Long-Term as well as Real-Time Monitoring of MOC Platforms

Real-time monitoring and analysis of cell metabolism are significant for evaluating the effects of a drug over an extended period [79,80,81]. Because of the labor-intensive demands and the complexity of the integration with low-volume bioreactors, conventional methods such as mass spectroscopy and enzyme-linked immunosorbent assay (ELISA) are inadequate to meet the needs of continual monitoring [82]. Miniature biomedical sensors appear as an effective tool to assess the dynamic metabolic process of living cells with high selectivity and sensitivity [83]. Incorporating biosensors into microfluidic devices contributes to the enhancement of sensing capabilities by improving the delivery of analytes [66]. Biosensors, originally used for the detection of some biomacromolecules; e.g., DNA [84,85], enzymes [86,87], peptides [88,89], and proteins [90,91], have now been widely used for different purposes when combined with microfluidic chips [92,93,94,95,96]. The utilization of these biosensors in the advancement of OOC platforms provides the ability for continual observation and analysis of chronic or retardant cellular responses to precise measurements of analytes or conditions in drug screening, disease modeling, and several other in vitro pharmacological or toxicological attributes [28,82].

**Figure 4:**
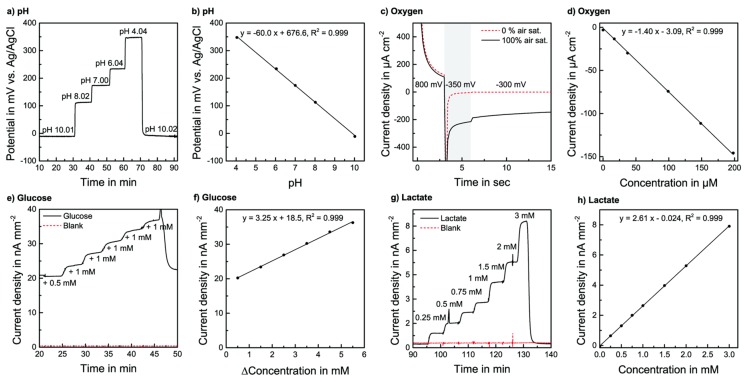
Sensor characterization in a cell culture medium at 37 °C at a flow rate of 2 μL·min^−1^: (**a**) Transient pH measurement. (**b**) Calibration for pH. (c) Current response of a 3-step chronoamperometric dissolved oxygen measurement protocol, with and without oxygen. (**d**) Calibration for dissolved oxygen. (**e**) Transient glucose measurement for glucose and a blank electrode, by spiking a medium containing glucose. (**f**) Glucose calibration with the blank signal subtracted. (**g**) Transient lactate measurement for lactate, and blank electrode in medium without FBS. (**h**) Lactate calibration with the blank signal subtracted. Reproduced from [79], with permission from the Royal Society of Chemistry, 2014.

Biochemical parameters in the microenvironment of the microfluidic platforms, such as changes in pH level and oxygen concentration, can be measured and read out by these efficient microsensor systems, which ensure consistently optimal physiological conditions and control over cell culture, as well as organoid behaviors [28,83,97]. However, it should be noted that the inappropriate extracellular acidity or oxygen tension may lead to undesirable variations in the physiology of the organoids, and lead to inappropriate detection accuracy of the OOC systems during drug screening studies [98,99,100,101,102]. Previously, some traditional electrode-based approaches were applied in this field for monitoring pH and oxygen. Nevertheless, they have now been replaced by low-cost optical sensing that is based on the detection of variations in the light absorption or fluorescent intensity of oxygen and pH indicators when the oxygen or pH of the microenvironment is changed, and this enables time-lapse studies to be conducted without interfering with the settings [103]. The optical sensing approach demonstrates the advantages of the construction of a compact and miniature detection device, compared with bulky spectrophotometry or microscopy technologies [104]. Biosensors for quantifying other parameters (e.g., glucose, lactate) used in this field are mostly based on the respective enzymes that are involved in their conversion. The first generation of biosensors had a fixed enzyme on a membrane, or a matrix located directly on the electrode. With the production of their respective by-products (e.g., H_2_O_2_), the enzyme (e.g., oxidase enzyme) oxidizes or reduces the analytes at a properly polarized electrode (e.g., platinum) [28]. Several attempts to incorporate pH and oxygen optical sensing systems and optical sensors for glucose and lactate into microfluidic platforms have been proposed [79,105,106,107,108,109,110,111]. In this framework, a microfluidic glass chip has been fabricated by combining cell culture and metabolic monitoring equipment with fully integrated biosensors. The pH and oxygen sensors showed a long-term stable, linear response in the cell culture area, and biosensors for lactate and glucose connected downstream by microfluidics exhibited linear, long-term stable, selective, and reversible behavior within the desired range [79] (Figure 4). This device provides a low-cost, easy-fabrication and convenient-operation analytical platform that can be applied in many microfluidic MOCs for continuous and real-time measurements of values of pH, dissolved oxygen levels, and concentrations of glucose and lactate, for drug screening in vitro. However, they have not been widely used in the metabolic detection of in vitro cell cultures compared to enzyme-based sensors.

**Figure 5:**
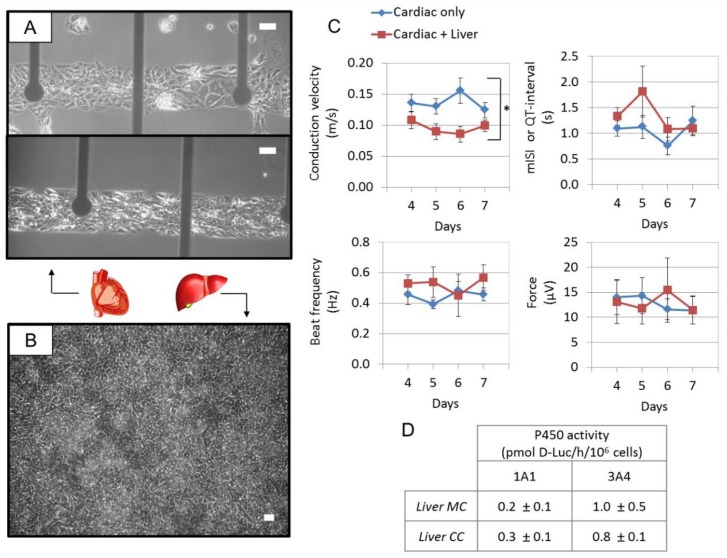
Characterization of the heart–liver system—serum-free and flow—with non-invasive measurements for seven days. Human cardiomyocytes and hepatocytes were studied over seven days in HSL3 medium. Representative morphology images are shown for human cardiomyocytes (**A**) in mono-culture (top) or co-culture (bottom) (80 μm scale) and hepatocytes in co-culture (**B**) after seven days in the housing (50 μm scale). Cardiac function was measured over seven days in the presence (red square) or absence (blue diamond) of hepatocytes. Cardiac function is plotted as conduction velocity, spontaneous beat frequency, mISI (or QT interval), and contractile force (**C**). Two-way ANOVA was performed to study the effects of culture time and the presence of the hepatocytes on the different cardiac functional parameters; conduction velocity (p = 0.8, 0.03), beat frequency (p = 0.8, 0.2), mISI (p = 0.3, 0.2) and force (p = 0.7, 0.9). Hepatic function was studied after seven days in the system with cardiomyocytes, and compared to the static mono-culture conditions. No significant differences were evident through a t-test for the 1A1 (p = 0.09) and 3A4 (p = 0.7) enzymes (**D**). For interpretation of the references to color in this Figure legend, the reader is referred to the Web version of this article. Reproduced from [120], with permission from Elsevier, 2018.

Incessant monitoring of cellular and micro-organ activities also plays a critical role in realizing the long-term testing of MOCs. The electrophysiological responses produced in cardiomyocytes and neurons have caused plenty of issues for emulating the environments of cardiac tissues and nerve tissues, and have affected the prediction of drugs in preclinical studies. In order to generate an appropriate electrochemical microenvironment, the mimicries of the heart and nerves are interconnected with electrodes [66]. Some conventional techniques, such as amperometry and patch-clamp, have been successfully used to evaluate the effects of drugs, with high sensitivity at the level of a single cell. However, they lack high-throughput screening, as they require intensive labor and limit the investigation of effects to a single pattern of action [112]. Alternatively, MEAs technology has been extensively used in electrophysiological experiments for monitoring the electrochemical signals in disease modeling of heart or nerve systems [113,114], drug testing [115], and toxicological models [116,117] in vitro, and it has been used to convey electric currents to cells in a process called microstimulation [118]. Different from intracellular monitoring techniques, this approach can shape non-invasive interfaces by directly contacting with cells, enabling quite a long window of time for recording cellular behaviors from active membranes of the cells and delivering electrical currents to stimulate the cells [119]. In a heart–liver multi-organ pumpless microfluidic system, Oleaga and colleagues used multi-microelectrode array chips, which were customized as two rows of five electrodes each, for monitoring electrochemical behaviors. Signals from the noninvasive interface between cells and the MEA chips were recorded with an amplifier to track the cardiac and hepatic functions [120] (Figure 5). This device enabled the long-term determination and more accurate predictions of xenobiotics toxicity, with lower costs in toxicology models, which emphasized the importance of the integration of MEAs technology in long-term testing of MOCs.

### 2.3. Multisensor-Integrated MOC Systems

**Figure 6:**
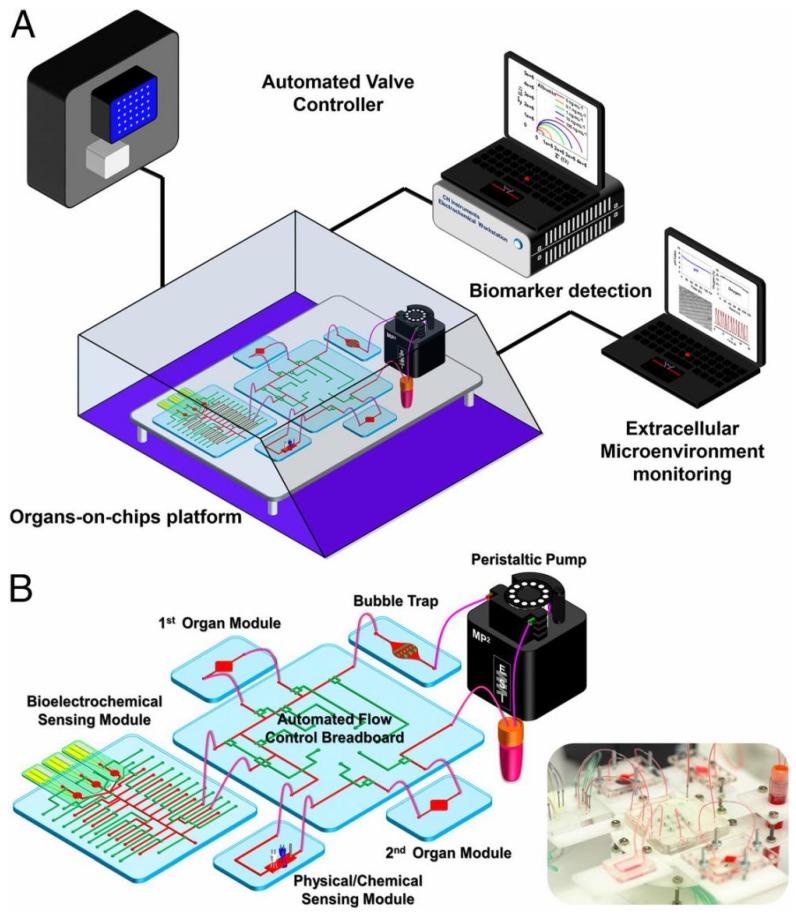
Integrated automated multiorgan-on-a-chip and sensing platform. (**A**) Schematic of a full system where the multiorgan-on-a-chip platform is encased in an in-house designed benchtop incubator, and of automated pneumatic valve controller, electronics for operating physical sensors, potentiostat for measuring electrochemical signals, and a computer for central programmed integration of all of the commands. (**B**) Schematic of the integrated microfluidic device consisting of modular components, including microbioreactors, breadboard, reservoir, bubble trap, physical sensors, and electrochemical biosensors. The inset shows the photograph of an integrated platform. Reproduced from [61], with permission from the Proceedings of the National Academy of Sciences of the United States of America, 2017.

As discussed above, multiple organoid models combining microfluidic technology with non-invasive biosensing systems offer several advantages over traditional models, such as better simulation of the physiology of human organs in vivo, and monitoring the biochemical attributes of these miniaturized organoid models in situ [121,122], while traditional analytical methods for the miniaturized MOC models are not suitable anymore, due to their large operating volumes and frequent system interference [121]. A large number of MOCs combining microfluidic technology with biomedical sensors to realize long-term testing of MOC platforms have been proposed. However, they are still limited in continually analyzing multi-organ interactions in situ and a lack of automated capability [61]. Thus, a system that seamlessly integrates various biomedical sensors into microfluidic multi-organ models, which can ideally work in an automated and continuous manner for a long period of time, is required. To address this limitation, Zhang and colleagues integrated a continuously and automatically operating sensing units that included a gold microelectrode set-based electrochemical immunobiosensors for capturing biomarkers, optical biosensors for monitoring microenvironmental parameters and microscopes for observing organoid morphologies and behaviors into a microfluidics-controlling breadboard based MOC system [61,123] (Figure 6). This platform with continual cell culture and different automated monitoring functions into microfluidic MOCs significantly enhanced the performance of long-term testing of drugs. Nevertheless, this approach of multisensor-integrated MOC platform using PDMS is not optimal as the applicability is limited due to absorption of hydrophobic small molecules and drugs by PDMS [61,124,125]. Further efforts on improving the fabrications and integrations of such multisensor-integrated microfluidic MOC models are very much desired to realize more efficient long-term testing and more accurate predictions of drug efficacy and toxic side-effects on MOCs for biomedical applications.

## 3. Biomedical Applications of Long-Term Testing in MOC Platforms

More often, the OOC systems are preferred over conventional approaches to precisely record drug performances with minimal resources in terms of time and cost, so that these models have gained popularity in the fields of therapeutic development over the past years [14,40,59,126,127]. In order to better mimic the interaction of in vivo human organs and the complex ADME process, several MOCs have recently emerged to displace the single OOC systems in biomedical applications. As listed in Table 1, there are four main proposed applications, including drug testing or toxicity studies, disease modeling, drug screening, and cancer metastasis. Among these studies, some lay more stress on the applications of long-term testing in MOC systems in therapeutic areas.

### 3.1. Proposed Biomedical Applications of Long-Term Testing in MOC Systems

#### 3.1.1. Drug Testing/Toxicology

The current process of drug development requires a high cost and an enormous lag of time. Moreover, about merely 10% of drug candidates entering clinical trials are finally approved [50]. Unpredictable issues such as severe toxicity or a lack of efficacy until the later stages of clinical trials are the main reasons for causing such a low efficiency rate; therefore, predicting drug toxicity earlier would save a lot of resources in terms of time and cost [128]. Plenty of efforts toward this goal have been proposed, for the application of in vitro multi-organ/tissue platforms into the field of drug testing/toxicology [128,129,130,131,133,142]. Particularly, in some studies, researchers have paid more attention toward long-term cultivation and continual testing by using MOCs [12,46,129,143]. In addition to the microfluidic four-organ chip based on a micropump for repeated dose systemic toxicity testing of drug candidates and in vitro observations of the ADME process over 28 days, which was introduced earlier [46], Materne et al. co-cultivated neurospheres and liver spheroids based on a microfluidic MOC platform containing a micropump, which ensured a stable long-term circulation of media to interconnect the two organ compartments over 14 days [132]. In this two-week toxicity assay with a substance exposure to the neurotoxic 2,5-hexanedione, the cytotoxicity results of such neurotoxins have shown dose-dependency. Similarly, an MOC platform designed with a peristaltic micropump and media reservoirs provided long-term co-culture of a human liver equivalent and a human skin biopsy. In this model, the liver microtissue displayed sensitivity to a diabetic drug, troglitazone, with liver toxicity at different molecular levels, which enabled repeated dose exposure of tissues to troglitazone for about seven days, fulfilling the potential for long-term systemic substance testing [12]. To further improve the performance of measuring real-time cellular functions with the maintenance of a cellular phenotype, an MOC device combining a microfluidic circuit with MEAs technology was used to investigate the effect of hepatic metabolism on off-target cardiotoxicity. By non-invasive monitoring of beat frequency, conduction velocity, QT-interval, and contractile force in two drug models related to cardiac side-effects dependent on hepatic metabolism, cyclophosphamide (CP), and terfenadine (TER), the system was validated, which allowed for long-term testing for the prediction of the cardiotoxicity transformation of drugs through hepatic metabolism [120].

#### 3.1.2. Disease Modeling

Another significant application of long-term testing using MOCs is disease modeling. Diseased tissues often show different responses to drugs, chemicals, or their metabolites, compared to healthy tissues [143,144]. In a multi-organ disease modeling system, the model organs capture human-specific features of a disease, which can enhance the authenticity of human pathophysiological responses and increase the effectiveness of the therapeutic strategies compared to conventional in vitro cell culture models and animal models [145]. To achieve long-term testing in an MOC disease model, Vunjak-Novakovic and coworkers established a multi-tissue platform with human-induced pluripotent stem cells (iPSCs)-based vascular, liver, and cardiac microtissues, which provided a faithful representation of the human vascular network, replicating metabolizing hepatic lobules and working myocardium for human biology research on health, injury, and disease over an extended period of time (about 28 days). The iPSCs used in this work provided a large variety of normal cells, and cells with genetic mutations for drug screening and disease modeling. The researchers also integrated biosensors into the iPSCs to monitor the ADME process and functional readouts for tissue cells in real time, which could offer profound insight into specific pathological mechanisms. This approach demonstrated its utility for predictions of physiological responses in the diseased microenvironment and the potential for the improvement of the translation of drug discovery [134].

### 3.2. Potential Applications of Long-Term Testing using MOCs

#### 3.2.1. Drug Screening

Due to the strong desire for a thorough and accurate in vitro assessment of drug potency in the processing of cancer therapeutics, multi-organ/tissue systems have emerged as a potential tool for drug screening [5,60,135,136]. In an attempt at anti-cancer drug screening by using an MOC system, a multi-tissue platform prepared by co-culturing HepG2/C3A (liver), MEG-01 (megakaryoblast, bone marrow), MES-SA (normal cancerous tissue), and MES-SA/DX-5 (multidrug-resistant cancer tissue) was designed to test the selective capability of a combination of drugs toward multidrug resistance (MDR) in cancer without major varieties in side-effects for acute exposure of 24 and 72 hr. The results illustrated the efficient prediction of the particular ability of chemotherapeutic/modulators mixtures that kill or reduce the growth of MDR tumor cells in vivo, with tolerable side-effects in normal tissues, demonstrating great potential for the screening of novel compounds [135]. However, almost all of the MOCs applied in this field could only test for a short period. To meet the screening requirements of drugs for chronic disease, long-term testing based on MOCs for drug screening are desired to detect the cellular response to drugs for chronic disease for an extended period.

#### 3.2.2. Cancer Metastasis

In addition to the several applications discussed above, another medical phenomenon that requires the use of more than one type of organ in the model is cancer metastasis. The discovery of novel anti-cancer and diagnostic tools [146] has been progressing steadily by revealing the specific signals of the cancer microenvironment and affecting the tumor growth, malignancy [147,148,149,150,151], and transvascular migration [152,153]. These multi-tissue metastasis-on-a-chip platforms contain multiple organoids that enable cancer cells to migrate from one site to another, to facilitate the detection of the dissemination of circulating tumor cells (CTCs), and their intravasation into capillaries during cancer metastasis [154,155]. Although some effort in this field has been made to observe cancer cell behaviors and to analyze tissue–tissue interactions in the physiologically relevant context by detecting variations of cancer cells, there are still only few attempts applying long-term testing in MOC systems into cancer metastasis modeling [137,138,139]. However, we believe that with the excellent properties mentioned above, the application of long-term testing in cancer metastasis modeling has the potential for better understanding of cancer biology and making significant progress with drug discovery in the future.

#### 3.2.3. Biomarker Detection

In the applications of MOCs, the determination and quantification of biomarkers or target metabolites are also key steps for analyzing biological reactions and the metabolism of drugs [156]. Often, the miniature-sized culture volume and the small number of cells in the MOC systems results in technical problems related to detection sensitivity. These issues have been solved by applying various analytical methods to biomarkers into the MOC models. As a conventional biosensing technology, enzyme-linked immunosorbent assay (ELISA) has been used in biomarker detection to assess the functions or conditions of cells in the MOCs [157]. In a recent study, an MOC system comprising lung tissues based on the PDMS model and bioprinted spherical liver and heart organoids, which were connected via a central fluid channel with fluid flow driven by a peristaltic micropump has been established, and the effects of bleomycin were determined by utilizing ELISA technology to quantify the levels of interleukin-8 (IL-8) and interleukin-1β (IL-1β) [140]. In addition, drug metabolites produced by cells provide complex and detailed information about organ responses, which have been explored by applying liquid chromatography coupled with a mass spectrometry (LC-MS) technology in MOC models to profile metabolomics in preclinical studies for high-throughput, better sample separation, high efficiency, and accuracy for measurement and diagnosis [157]. Satoh and coworkers developed an LC-MS system-coupled MOC platform containing tissues of liver, intestine, cancer, and connective cells that were interconnected via microchannels with medium driven by a sequential pneumatic-pressure-control system to detect the concentrations of capecitabine and 5-fluorouracil (5-FU) in the medium [141]. These models on the application of MOC systems in biomarker detection enabled the determination of in vivo information, based on variations in the composition and concentration of analytes in the medium [157]. Thus, we believe that as technologies based on biomarker detection and long-term testing using MOCs advance, efforts at long-term testing in MOCs towards this application to analyze the chronic organ responses and the long-term pharmaceutical metabolism will be shortly become substantially feasible.

#### 3.2.4. Personalized Medicine

A large number of MOC systems in vitro have so far been applied in the area of therapeutic development, but these therapies are primarily designed for the average patient, with no consideration of people’s diversity [158]. Prescribed treatments are usually based on the general success rate of drugs, rather than the response of specific patients to drugs; thus, efficient tools in support of predicting how a specific individual responds to a drug are greatly needed [155,159,160]. Microfluidic MOC platforms containing cells taken from patients are currently emerging as a new tool for developing personalized medicines. As described above, long-term testing in MOCs enables a better biomimicking microenvironment, and the ability for continual observations of the ADME process of drugs, and more accurate predictions of responses by multiple organs, gives us confidence that this approach will be possible for defining appropriate drugs and dosages for individual patients before treatment, and this will greatly change patient care and improve treatments for cancer and other diseases [155,158].

## 4. Conclusions and Perspectives

MOCs can emulate the key aspects of an in vivo human environment, and they are capable of mimicking the organ–organ interactions and the complex ADME process. In this review, we have discussed the advancements of realizing long-term testing of MOCs, which are greatly desired for enhancing the capability of analyzing multi-organ interactions more accurately and stably, for better detection of chronic cellular reactions in the human body. The first effort for this goal has been achieved by using microfluidic technology to provide a steady and sustained flow of culture medium, and to connect various organ compartments for a long-term multi-tissue co-culture approach. Then, incorporating biomedical sensors into MOC systems provides low-cost and convenient-operation analytical platforms for the detection of microenvironmental parameters and electrophysiological responses for real-time monitoring of MOC platforms. Finally, designing multisensor-integrated microfluidic MOC systems promotes the performance of automated and continuous monitoring of the metabolic processes of drugs and the conditions of the MOC systems. However, there are still a few limitations in the proposed experiments, and further efforts on the integration of multisensor systems in the future are still required to address these issues. In addition, we gave a brief overview of various proposed biomedical applications of long-term testing in MOCs, mainly in drug testing/toxicology and disease modeling. These applications of MOCs showcased the potential for predicting efficacy and toxic side-effects with higher accuracy, to promote the development process of drugs and the discovery of novel therapeutic strategies. Moreover, we introduced various potential applications for long-term testing in MOC systems in drug screening for chronic disease, cancer metastasis modeling for better understanding cancer biology, and making good progress in drug discovery, biomarker detection for the analysis chronic organ responses and long-term pharmaceutical metabolism and personalized medicine for more accurate predictions for specific individual responses to a drug, to improve the treatment of cancer and other diseases.

## Figures and Tables

**Table 1:** Biomedical applications of MOC platforms.

Application	Multi-Organ/Tissue System	Fabrication Approach	Outcome	References
**Drug testing/toxicology**	Liver, tumor, and marrow	This model combined a three-compartment microscale cell culture analog (µCCA) device exposed to a pumpless gravity-induced flow with a mathematical pharmacokinetic and pharmacodynamic (PK-PD) model.	This model promoted the analysis and prediction of the effects of 5-fluorouracil (5-FU).	[128]
	Liver, intestine, skin, and kidney	This model integrated two peristaltic on-chip micropumps and microfluidic channels connecting four tissue culture chambers for two microfluidic circuits into the four-organ-chip.	This model was helpful for repeated dose toxicity testing of drug candidates and further in vitro absorption, distribution, metabolism and elimination (ADME) observation.	[46]
	Liver, colorectal tissues	These models cultured spherical microtissues in parallel, connected by a microfluidic-channel network, with liquid flow controlled through a hanging-drop device.	These models were helpful for testing drug effects at different concentrations.	[129,130,131]
	Liver, nerve tissues	This model connecting two tissue compartments exposed by microfluidic channels was maintained in a combined media circuit.	This model showed the dose-dependent cytotoxicity result of the neurotoxic compound 2,5-hexanedione.	[132]
	Liver, heart	This model contained human-induced pluripotent stem cells (iPSCs)-derived liver and heart tissues, which were exposed to serum-free medium flow using a pumpless system.	This model was helpful for the prediction of the cardiotoxicity transformation of drugs through hepatic metabolism.	[120]
	Liver, skin tissues	This model used a single polydimethylsiloxane (PDMS) layer integrating the respectively arranged channels interconnecting the tissue counterparts, peristaltic on-chip micropumps, media reservoirs, and openings for culture compartments.	This model tested the liver toxicity of troglitazone at different molecular levels.	[12]
	Lung, gut, skin, vascular, liver, and kidney	This model, using physiologically-based pharmacokinetics with pharmacodynamic (PBPK/PD) models for estimating ADME parameters, was made of PDMS and microfluidic channels for connecting different organ compartments.	This model was helpful for PBPK/PD modeling and drug development in different stages.	[133]
**Disease modeling**	Liver, heart, and vascular system	This model interconnected iPSCs-derived cardiomyocytes and hepatocytes by 3D-printed rigid filament networks of a carbohydrate glass with endothelial cells, and perfused the networks with high-pressure pulsatile blood flow.	This model was helpful for predictions of physiological responses in the diseased microenvironment.	[134]
**Drug screening**	Liver, heart, lung, and kidney	This model adopted allometric scaling for coupled non-linear organ-on-a-chip (OOC)/ multi-organ-on-chip (MOC) systems to create micro-organs maintained by a universal media.	This model was helpful for the screening of new drugs for efficacy and potential side-effects	[60]
	Liver, marrow, megakaryoblast, and cancerous tissues	This model integrated a µCCA device into a silicon chip, on which four functional tissues were cultured in corresponding chambers connected by Pharmed tubing, with recirculating flow being provided by a peristaltic pump.	This model was helpful to predict the selectivity of chemotherapeutic/modulator mixtures for killing or reducing the growth of multidrug resistance (MDR) tumor cells in vivo.	[135]
	Liver, intestine, and breast carcinoma cells	This model containing microtissues of liver, intestine and the breast carcinoma cells cultured in the target components consisting of a slide and PDMS layers, having microchannels made by photolithography.	This model was helpful for the evaluation overall properties of orally ingested drugs, foods, and chemicals.	[136]
**Cancer metastasis**	Marrow, mesenchymal stem cells, and breast cancer cells	This model bonded a bored PDMS layer to a cover glass to create microfluidic channels with oxygen plasma treatment, and provided eight cell-culture gel regions connected to the central media channel.	This model was helpful to mimic the dissemination of breast cancer cells into bone.	[137]
	Brain, bone, liver, and lung carcinoma cells	This model combined three PDMS sheets and two thin PDMS microporous membranes to create three parallel microchannels connecting an upstream micro-lung and three downstream micro-organs.	This model was helpful for observing lung cancer cell behaviors in a physiologically relevant context.	[138]
	Intestine, liver, and colon carcinoma tissues	This model, comprising two independent cell-culture chambers connected by a circulating fluid flow, was fabricated with a hyaluronic acid-based hydrogel system in which the metastatic colon carcinoma tumor foci were created.	This model was helpful for studying the process of the migration of colon carcinoma cells.	[139]
**Biomarker detection**	Heart, liver, and lung	This model comprised lung tissues based on the PDMS model and bioprinted spherical liver and heart organoids, which are connected via a central fluid channel with fluid flow driven by a peristaltic micropump.	This model was helpful to utilize enzyme-linked immunosorbent assays (ELISAs) to determine the effect of bleomycin to quantify the levels of interleukin-8 (IL-8) and interleukin-1β (IL-1β).	[140]
	Liver, intestine, cancer, and connective cells	This model contained two culture chambers interconnected in each culture unit via microchannels with a medium driven by a sequential pneumatic pressure-control system.	This model was helpful for liquid chromatography coupled with a mass spectrometry (LC-MS) system, to measure the concentrations of capecitabine and 5-FU in the medium of the model.	[141]
